# *PLoS Medicine* and Publication Ethics: When Is It Research?

**DOI:** 10.1371/journal.pmed.0040169

**Published:** 2007-04-24

**Authors:** Mary Ann Baily

I read this editorial with interest [1]. The issues discussed under the heading “Is a Program Description a Research Paper?” are closely related to the subject matter of a recently completed Hastings Center project funded by the Agency for Healthcare Research and Quality.

The project was carried out by a group of distinguished participants with expertise in medicine, law, nursing, quality improvement methods, research ethics, medical editing and publishing, health services research, and health policy and regulation. It addressed the ethics of using quality improvement (QI) methods to improve health care quality and safety, and we spent considerable time on the vexing question of when (if ever) QI activities meet the regulatory definition of research and should be submitted to an Institutional Review Board (IRB) for ethical review. We also discussed the relationship between publication and IRB review.

Our analysis would lead to the same conclusion that was reached in the editorial: that the particular case example was not research and did not require IRB review. Readers might find the report's reasoning on the issues interesting, however—and also useful in assessing other cases.

Both the project report [2] and a book of background papers [3] have been published, and they can be downloaded in PDF form at no charge from the Web site of The Hastings Center (http://www.thehastingscenter.org) or the Institute for Healthcare Improvement (http://www.ihi.org).
